# Diagnostic AI and Cardiac Diseases

**DOI:** 10.3390/diagnostics12122901

**Published:** 2022-11-22

**Authors:** Dilber Uzun Ozsahin, Cemre Ozgocmen, Ozlem Balcioglu, Ilker Ozsahin, Berna Uzun

**Affiliations:** 1 Medical Diagnostic Imaging Department, College of Health Sciences, University of Sharjah, Sharjah 27272, United Arab Emirates; 2 Operational Research Center in Healthcare, Near East University, TRNC Mersin 10, 99138 Nicosia, Turkey; 3 Department of Biomedical Engineering, Faculty of Engineering, Near East University, TRNC Mersin 10, 99138 Nicosia, Turkey; 4 Department of Cardiovascular Surgery, Faculty of Medicine, Near East University, TRNC Mersin 10, 99138 Nicosia, Turkey; 5 Brain Health Imaging Institute, Department of Radiology, Weill Cornell Medicine, New York, NY 10065, USA; 6 Department of Statistics, Carlos III University of Madrid, 28903 Madrid, Spain; 7 Department of Mathematics, Faculty of Sciences and Letters, Near East University, TRNC Mersin 10, 99138 Nicosia, Turkey

**Keywords:** artificial intelligence, machine learning, cardiac disease, diagnosis

## Abstract

(1) Background: The purpose of this study is to review and highlight recent advances in diagnostic uses of artificial intelligence (AI) for cardiac diseases, in order to emphasize expected benefits to both patients and healthcare specialists; (2) Methods: We focused on four key search terms (Cardiac Disease, diagnosis, artificial intelligence, machine learning) across three different databases (Pubmed, European Heart Journal, Science Direct) between 2017–2022 in order to reach relatively more recent developments in the field. Our review was structured in order to clearly differentiate publications according to the disease they aim to diagnose (coronary artery disease, electrophysiological and structural heart diseases); (3) Results: Each study had different levels of success, where declared sensitivity, specificity, precision, accuracy, area under curve and F1 scores were reported for every article reviewed; (4) Conclusions: the number and quality of AI-assisted cardiac disease diagnosis publications will continue to increase through each year. We believe AI-based diagnosis should only be viewed as an additional tool assisting doctors’ own judgement, where the end goal is to provide better quality of healthcare and to make getting medical help more affordable and more accessible, for everyone, everywhere.

## 1. Introduction

As it is with other parts of our body, there are many things that can go wrong with our heart. Structural heart defects at birth, lesions in blood vessels, heart valve calcification over time, heart muscle inefficiency for various reasons and electrical signal conduction abnormalities are all examples of cardiac diseases anyone can experience throughout their lifetime.

The Centers for Disease Control and Prevention (CDC) statistics show that 20% of deaths in U.S.A. were caused by cardiac diseases in 2020. This amounts to 696,962 people and it is the leading cause of death in the country [1]. Although cardiac diseases are much more prevalent with elderly patients, it is statistically relevant for all age groups, depending on risk factors present in a patient’s life. Therefore, it is easy to understand the amount of research that goes into understanding, diagnosing and treating cardiac diseases. By extension, it is also inevitable that artificial intelligence (AI) research also crosses its path with cardiac disease diagnosis, where earlier and non-invasive diagnosis of as many patients as possible can be seen as the ultimate goal, saving many lives in the process.

Artificial intelligence (AI), first elaborated by Alan Turing in 1950 [2], is the concept of creating a digital mind that can learn, adapt, react and “think” in a similar manner as a human being. Machine learning (ML) is a process that is closely linked to the concept of AI, where a computer model is enabled to learn new skills and information, and as a result, it can provide useful feedback and perform tasks where classical algorithm-based computer programming falls short. In theory, it is possible for an AI to perform any task imaginable as long as the ML process is sufficiently advanced and robust [3,4].

There are two main approaches to machine learning with many more under each archetype. First is “supervised machine learning”, where a dataset is introduced to the model with each case labelled with a class. Computer model then finds commonalities between each case that belongs to the same class and thus “learns” to identify any new unlabelled case as belonging to one of those classes. Supervised ML is especially useful if the task at hand requires the input data to be sorted into predetermined classes or making predictions. However, labelling the dataset for training requires expert knowledge and is time consuming [5,6].

**Figure 1:**
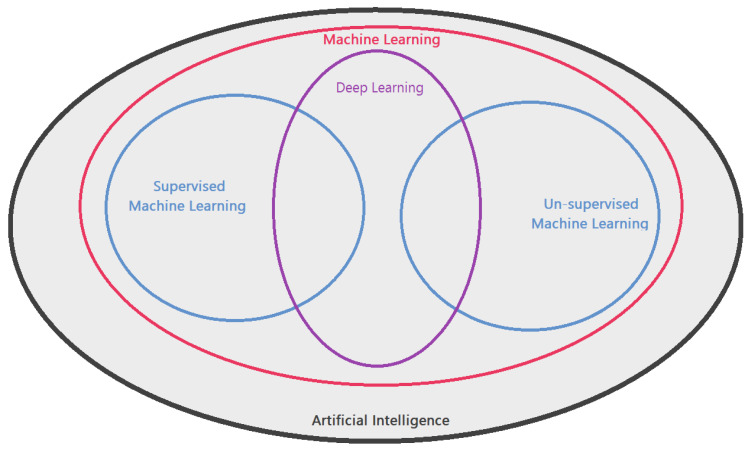
A Venn diagram visually explaining AI and machine learning relationship.

The second approach of “unsupervised machine learning” involves giving the computer model all the data without telling the model which cases belong to which class, and letting it make connections itself where it will sort cases into different classes according to the connections it made while learning. Unsupervised ML can be extremely useful in gaining new understanding about complex systems with many variables as it will make clustering decisions and feature associations by itself, sometimes surprising the experts of the field. However, database size required by an unsupervised ML model is comparatively much larger, where sometimes building the database itself becomes an issue on its own [5,6]. Figure 1 shows the diagram of the AI and machine learning relationship.

AI can become extremely useful for healthcare in the near future, as it is already becoming so for most other industries and our everyday lives. As healthcare is becoming more and more digitized and interconnected, productive integration of AI systems into standard hospital care is rapidly becoming inevitable. AI can improve many aspects of patient care like medical imaging quality, early diagnosis, prognosis prediction, risk stratification, patient data analysis, personalized treatments and more. For example, as compact devices and biosensors become more available and integrated in our lives, remote monitoring of vitals for the whole population or at least some specific risk groups will be discussed and this will only be feasible with AI shouldering the immense volume of data analysis necessary [7,8].

Additionally, physicians and other healthcare professionals are treating more and more cases per day as the population grows, life expectancy increases and as technology required to treat previously unmanageable diseases becomes more and more available. In order to alleviate their workload, which is already much higher than many other fields of work, and avoid related mistakes that are inevitable, AI assistance will become indispensable. Instead of losing precious time, energy and hospital budget on tasks that can be relegated to AI, physicians and other healthcare professionals can focus on their patients [9,10].

Purpose of this study is to review and highlight recent advances in diagnostic uses of artificial intelligence (AI) for cardiac diseases, in order to emphasize expected benefits to both patients and healthcare specialists, as the future of AI and machine learning (ML) is to be integrated in all aspects of our lives, especially in medicine.

Towards that goal, we will be highlighting some noteworthy challenges relevant to this topic like computing power limitations, data set availability in medical fields, inefficient research targets, and some future directions like ethical/legal considerations that may arise and the idea for a multi-national network of an integrated healthcare system.

## 2. Method

In this review, we focused on four key search terms (cardiac disease, diagnosis, artificial intelligence, machine learning) across three different databases (Pubmed, European Heart Journal, Science Direct). As per our aim, we limited our search to publications between 2017–2022 in order to reach relatively more recent developments in the field.

There were 346 results in total for original articles with keywords mentioned above, after review articles, case reports and meta-data analysis articles were filtered out. Looking into the content of each publication, we identified that some of the results were not related to cardiac diseases and/or artificial intelligence. Among those that were, some of the results were about disease/feature classification and predictive risk stratification, not diagnostic works, further reducing the number of diagnosis relevant publications. In the end, we identified 38 publications that are relevant to our review.

We elected to subcategorized these publications under the results section in relation to the disease it aims to diagnose in order to provide a more comprehensive comparison and a more coherent reading experience for readers.

A flowchart detailing the process of how relevant publications are obtained, classified and explored can be found below.

Although machine learning methods utilized in each publication will be mentioned, the primary focus of this review will be on medical resources utilized, diagnosed condition and resulting success rates.

## 3. Results

### 3.1. Structural Heart Diseases

Hsiang et al. published an article in 2022 about using chest X-rays in order to identify a left ventricular ejection fraction (LVEF) that is lower than 35%. They used 77,227 chest X-rays to train a deep learning model and another 13,320 for testing. The area under curve (AUC) for detection of LVEF < 35% was found to be 0.867. Furthermore, patients with higher than 50% LVEF that were marked by the AI model were found to be at high risk for developing low LVEF in the future according to currently accepted risk assessment guidelines [11].

Salte et al. investigated the possibility of using echocardiography to automatically measure global longitudinal strain (GLS) in 2021. A total of 200 patients were examined in this study and their data were used to train a deep learning ML model. The process of GLS measurement, which was reported to be underused in clinical practices for the amount of time it takes to perform, only took 15 s for each patient. The proposed system could perform the measurement on 89% of the patients, where GLS was −12.0 ± 4.1% for the AI method and −13.5 ± 5.3% for the reference method [12].

Bahado-Singh et al. were interested in analyzing cell-free DNA that is present in maternal blood in order to identify fetal congenital heart defects. In 2022, they published that by identifying altered gene pathways that result in congenital heart defects, with the help of AI, they can determine various heart defects that will develop with fetus. A total of 6 different AI systems, like random forest, support vector machine and deep learning were used for cross validation since data set size was small (12 cases and 26 controls). Using a combination of nucleotide markers, they achieved an AUC of 0.97, sensitivity of 98% and specificity of 94% [13].

Cheema et al. sought to combine AI with an integrated health system where 11 hospitals are, patient data-wise, interconnected. These data include lab results, demographics, prescription information, procedure codes and more. A total of 7346 patients were identified within the health system to have either stage C or stage D heart failure (HF), and 7500 patients were chosen to be the control group as a result of their normal echocardiography. Using a deep learning algorithm, they were able to classify each case as belonging to one of three categories (stage C, D, healthy) with an overall accuracy of 83% compared to 75% accuracy of physician assessment [14].

Shrivastava et al. discussed how it is possible to diagnose dilated cardiomyopathy using AI with 12-lead ECG instead of an echocardiography in their 2021 publication. In total, 421 dilated cardiomyopathy patients and 16,025 control patients with normal LVEF were used as the database with AUC 0.955, sensitivity of 98.8%, and specificity of 44.8%. The negative predictive value was at 100% while positive predictive value was 1.8% where the reported conclusion was that it is a cost-effective screening tool [15].

Kwon et al. investigated the use of ECG data in 2020 in order to diagnose mitral regurgitation in patients. With a database of 56,670 training ECGs, 3174 internal and 10,865 external test ECGs, proposed AI model identified the P-wave and T-wave as the high weight features. This study produced an AUC of 0.816 for internal testing and 0.877 for external testing [16].

Jentzer et al. used ECG signals in order to identify LV systolic dysfunction for ICU patients in 2021. A study involving 5680 patients, AI was used to obtain 0.83 AUC and overall accuracy of 76% [17].

Lee et al. published an article in 2022 about detection of cardiomyopathy in the peripartum period using ECG signals. Utilizing a deep learning model, AI first learned to identify LV systolic dysfunction using 122,733 ECG samples. For external validation, 271 ECGs of pregnant women were used, producing a result of AUC 0.877, sensitivity 0.833, specificity 0.809, PPV 0.352 and NPV 0.975 [18].

Thalappillil et al. were looking to replace CT aortic annulus measurements for TAVI procedures with AI backed echocardiography. A total of 47 patients implanted with a new heart valve were included in this study. Comparing AI measurements with CT measurements, there was a −4.62 to 1.26 mm difference for derived area and −4.51 to 1.45 mm for the derived perimeter value [19].

Liu et al. used ECG and transthoracic echocardiography (TTE) together in order to diagnose pulmonary hypertension. In their 2022 work, they utilized a deep learning model with 10-fold cross-validation neural network, taking advantage of 41,097 patient data. Results show that AUC was 0.88 with 81.0% sensitivity and 79.6% specificity [20].

Sun et al. used 12-lead ECG and TTE in order to identify patients with <50% LVEF in 2021. A total of 21,732 data pairs trained a CNN deep learning model, while 2530 were used for testing. The ML model produced an overall accuracy of 73.9%, sensitivity of 69.2%, specificity of 70.5%, positive predictive value of 70.1%, and negative predictive value of 69.9% [21].

Thompson et al. published an article in 2019 that demonstrated valvular or congenital heart disease diagnosis could be made using AI assisted auscultation of heart murmurs. In the database obtained from Johns Hopkins Cardiac Auscultatory Recording Database, they wanted to classify each case into either pathological murmur, innocent murmur or no murmur classes. Using 3180 heart sounds, they were able to achieve 93% sensitivity, 81% specificity and 88% accuracy [22].

Harmon et al. used 12-lead ECG in order to detect LV systolic dysfunction with a CNN deep learning model. In total, 44,986 patients, who had an echo pair for independent verification, were used in this study and testing phase was done with 52,870 patients. With a 0.93 AUC, the AI model could detect EF < 35% [23].

Makimoto et al. investigated the use of auscultatory data in diagnosing severe aortic valve stenosis in 2022. By using the sound data that were reported to be cheaper and faster to acquire, they hoped to reduce the percentage of patients that goes undiagnosed. Using three separate CNNs with five-fold cross-validation, they trained the AI models with three different hospital data first separately for each location and then all data together. Comparing each result in order to find the best ML system for the task, they later exported the system to a smart phone as an application where it achieved a 97.6% sensitivity, 94.4% specificity, 95.7% accuracy, and F1 value of 0.93. Compared with the consensus of cardiologists, these results were 81.0%, 93.3%, 89.4% and 0.829, respectively [24].

Attia et al. sought to use a digital stethoscope in order to detect patients with low EF in 2022. Their aim is to significantly reduce the asymptomatic low EF patient numbers that might cause serious health issues along the line, which currently stands at a reported 8% of population. The conceived value of this study lies at diagnosis using a relatively very simple device that obtains 1-lead ECG signals and sound recordings. To accomplish this, they used a CNN based ML model to classify EF < 35%, EF < 40%, EF < 50%. Results were AUC 0.91 for EF < 35%, 0.89 for EF < 40% and 0.84 for EF < 50% [25].

Ghanayim et al. developed an electronic stethoscope which was able to record infrasound. They used this device to obtain heart sounds from 100 patients. Using an undisclosed AI structure, they were able to differentiate mild or severe aortic stenosis and no aortic stenosis classes. Their declared results in 2022 were 86% sensitivity and 100% specificity in testing phase. Validation group scored 84% sensitivity and 92% specificity while additional testing group had 90% sensitivity and 84% specificity [26].

Ueda et al. used chest X-rays in 2021 in order to detect aortic stenosis. Training three different deep learning models with 10,433 chest X-rays, binary classification of aortic stenosis positive or negative classes was prepared as output. Instead of using the best performing DL model out of the three, all of them were used simultaneously via a voting-based ensemble as it produced the best results. Looking at the final performance 0.83 AUC, 0.78 sensitivity, 0.71 specificity, 0.71 accuracy, 0.18 positive predictive value and 0.97 negative predictive value were achieved [27].

### 3.2. Electrophysiological Heart Diseases

Nakamura et al. looked into identifying premature ventricular complex (PVC) origin locations using 12-lead ECG in 2021. They used ML with two different methods to train the model, first of it being a support vector machine (SVM), and the second was a convolutional neural network (CNN) in order to classify a PVC’s location of origin. They used four basic class groups for the AI to consider, which were left, right, outflow tract and others. They wanted to compare their ML model with electrophysiologists and another algorithm in order to measure their success. They reported obtaining the following accuracies: SVM 0.85, CNN 0.80, electrophysiologists 0.73, and existing algorithm 0.86 [28].

Chen et al. were interested in investigating if a wearable monitoring device that can record photoplethysmographic (PPG) data and single-channel ECG data can be used in order to detect atrial fibrillation (AF) presence in a patient. Their 2020 publication shows that using a deep convolutional neural network, they were able to measure wristband PPG classification performance as 88% sensitivity, 96.41% specificity and 93.27% accuracy. Wristband ECG performance was 87.33%, 99.20% and 94.76%, respectively. Comparing these results with how physicians performed, their sensitivity, specificity and accuracy were 96.67%, 98.01%, and 97.51%, respectively [29].

Sau et al. used a CNN deep learning model in 2022 in order to distinguish between atrial tachyarrhythmias that can be cured with a cavotricuspid isthmus ablation, namely atrial flutter (AFL), and others atrial tachyarrhythmias. In this binary classification endeavour, they used 5 s 12-lead ECG recordings for each patient and achieved an accuracy of 86% versus median electrophysiologist accuracy of 79% [30].

Jo et al. used 12-lead ECG recordings of patients in 2021 in order to identify paroxysmal supraventricular tachycardia during sinus rhythm. Using data from a total of 12,955 patients, the Deep learning ML model was trained and tested. At the end of the study, research group was able to show results of 0.970 accuracy, 0.868 sensitivity, 0.972 specificity, 0.255 positive predictive value and 0.998 negative predictive value [31].

Chang et al. used an ML model with a recurrent neural network structure in 2021 in order to classify 12-lead ECG data into 13 arrhythmia classes (ST-elevation MI, AF, AFL, Atrial premature beat, ventricular bigeminy, complete heart block, ectopic atrial rhythm, first-degree AV block, sinus rhythm, paroxsysmal SVT, second-degree AV block, sinus tachycardia, PVC). A total of 60,537 ECG recordings from 35,981 patients were used and, as a result, achieved a performance of 0.987 accuracy and 0.997 area under curve [32].

Au-Yeung et al. published an article in 2021 that attempted to develop a heart rhythm monitoring and an alert system for ICU using a PhysioNet database. They utilized a random forest classifier based supervised ML model in order to evaluate ECG, blood pressure and PPG data and they found out that they could discriminate between eight classes (six arrhythmias) with a sensitivity of 81.54% [33].

Lee et al. proposed a deep learning model in 2022 that can diagnose enlarged atrium from exercise ECG recordings that can lead to AF. Using a convolutional recurrent neural network, they were able to perform a binary categorization with an unspecified performance [34].

Pandey et al. utilized an ensemble-based support vector machine classifier using the arrhythmia database of MIT-BIH. Their aim was to classify ECG recordings into one of four classes of normal rhythm or arrhythmia (SV ectopic beat, PVC, fusion) using only four features (wavelets, high order statistics, R-R intervals, morphological features). They were able to produce a result of 94.4% accuracy [35].

Zhu et al. published an article in 2020 where their aim was to diagnose a patient ECG using ML. They employed a CNN ML model using 12-lead ECG data in order to differentiate 21 rhythms (normal, sinus tachycardia, sinus bradycardia, premature atrial contraction, atrial rhythm, atrial tachycardia, atrial flutter, atrial fibrillation, premature junctional contraction, junctional rhythm, paroxysmal supraventricular tachycardia, premature ventricular contraction, idioventricular rhythm, ventricular tachycardia, artificial atrial pacing rhythm, artificial ventricular pacing rhythm, left bundle branch block, first-degree atrioventricular block, Mobitz type I second-degree atrioventricular block, Wolff–Parkinson–White syndrome type A, and Wolff–Parkinson–White syndrome type B). Training with 135,817 ECG recordings and testing with 17,955 ECGs, they were able to outperform most physicians with an F1 score of 0.887 compared to 0.789–0.831 mean F1 scores for physicians [36].

### 3.3. Coronary Artery Disease

Otaki et al. published an article in 2022 that seeks to identify coronary artery disease in a patient using data obtained from SPECT images. With a dataset of 3578 patients, they trained a deep learning model where stress myocardial perfusion, wall motion, and wall thickening map, left ventricular volume, age and sex were selected as model input features. After integrating their system to a general-purpose clinical workstation, they obtained an AUC of 0.83 which was significantly higher than their compared results (automatic stress total perfusion deficit 0.73, reader diagnosis 0.65) [37].

Braun et al. utilized a supervised ML model in order to detect clinically asymptomatic coronary artery disease in 2020. With five lead vectorcardiography and a 595-patient database, they achieved a sensitivity score of 90.2 ± 4.2% for female patients and 97.2 ± 3.1% for male patients, specificity of 74.4 ± 9.8% and 76.1 ± 8.5% for females and males, respectively. Overall accuracy was 82.5 ± 6.4% for female and 90.7 ± 3.3% for male patients [38].

Zhao et al. used ECG signals in order to automatically identify ST segment elevated MI and regain precious time lost between identification and treatment time delay. Published in 2020, 667 ST elevated MI patient ECG data and 7571 control ECG data were used in order to train the ML algorithm. The trained system showed AUC of 0.9954 with sensitivity of 96.75%, specificity of 99.20%, accuracy of 99.01%, precision of 90.86% and F1 score of 0.9372 [39].

Choi et al. proposed an inventive idea in 2022 where they used an image of ECG recordings in their dataset instead of the electronic signal recording in order to detect ST elevated MI. Their reasoning was that a mobile phone with a camera is extremely accessible for everyone. With an undisclosed image-based ML model, they completed the training phase with 187 patient recordings where 96 of patients were known to be diagnosed with ST elevated MI. The AUC of proposed ML model was 0.919 where for it was 0.843 for emergency physicians and 0.817 for cardiologists [40].

Cho et al. used intravascular ultrasound (IVUS) images with a deep learning model in 2021 with the aim of identifying and classifying plaque characteristics present in a cardiac blood vessel. A total of 598 IVUS image sets were used in this study with a total of three classes, which are calcified plaque, attenuated plaque and none. The proposed model was able to achieve attenuation sensitivity of 80%, specificity of 96%, accuracy of 93% and calcium sensitivity of 86%, specificity of 97% and accuracy of 96%. Compared to human performance, per-vessel analysis achieved similar results (0.95 human, 0.89 ML model), producing these results in 7.8 s [41].

Stuckey et al. developed a supervised ML model using linear regression in 2018 in order to assess coronary artery disease presence. They acquired phase signals from CT for a total of 606 patients, just before the planned coronary angiography, which was used to produce labels for the supervised training model. A total of 512 patient data points were used for training and 94 patient data points were used for testing stage. The study produced a result of 92% sensitivity, 62% specificity, 46% positive predictive value and negative predictive value of 96% [42].

Cho et al. published an article in 2019, aiming to assess fractional flow reserve (FFR) using coronary angiography. A supervised machine learning model that evaluates intermediate lesions was built in order to classify the data into FFR > 80 or FFR < 80. With a dataset of 1501 patients, achieved results were 78 ± 4% diagnostic accuracy and 0.84 ± 0.03 AUC. Out of 24 features used in ML model, 12 of them were found to be high ranking, including segment, body surface area, distal lumen diameter, minimum lumen diameter and length of lumen. Using only these 12 high ranking features, they were able to achieve 81 ± 1% diagnostic accuracy and 0.87 ± 0.01 AUC [43].

Lee et al. predicted that they can assess coronary artery disease presence using a treadmill exercise test (TET). In a 2021 publication, they discussed using 93 features with five different ML models (random forest, logistic regression, support vector machine, k-nearest neighbour, extreme gradient boosting). Among these features, exercise performance, hemodynamics and ST-segment changes, comorbidity, smoking, Framingham risk score, height and weight were present. Out of the five different ML models, random forest showed the best performance, AUC of 0.74, sensitivity of 85% and false positive rate of 55% compared to the 76.3% of conventional TET [44].

Lipkin et al. proposed that they could use coronary CT angiography (CCTA) with AI-based quantitative CT (QCT) in order to produce a better detection rate than myocardial perfusion imaging (MPI) for obstructive stenosis. Using a pre-built cloud based QCT software on acquired CCTA data, they were able to outperform MPI on obstructive stenosis detection across two classes with AI-QCT. AUC scores for stenosis > 50% showed 0.66 for MPI vs. 0.88 for AI-QCT, stenosis > 70% showed 0.7 for MPI vs 0.90 for AI-QCT. Publication suggests that CCTA with AI-QCT outperforms MPI and thus it could reduce the number of patients undergoing invasive tests and reduce healthcare costs [45].

Kurata et al. utilized coronary computed tomography-derived computational fractional flow reserve (CT-FFR) in 2019 in order to detect coronary artery disease. With a dataset of 74 patients, they employed a prototype ML model (cFFR version 3.0.0, Siemens Healthcare, Tokyo, Japan) comparing CCTA and CT-FFR performance. Obtained results show that FFR < 0.8 CT-FFR had AUC of 0.907 where CTA stenosis > 50% had 0.595 and stenosis > 70% had 0.603. CT-FFR had an analysis time of 16.4 ± 7.5 min [46].

Tang et al. used CCTA derived FFR in 2019 in order to detect lesion-specific ischemia. They adopted an ML algorithm called cFFR_ML_ and obtained CT-FFR values from 136 patients across four healthcare centers. Invasive FFR measurements were used as reference. Study revealed that cFFR_ML_ had 0.85 sensitivity, 0.94 specificity and 0.90 accuracy versus 0.95 sensitivity, 0.28 specificity and 0.55 accuracy for CCTA [47].

Choi et al. published an article in 2021 where they compare coronary artery disease assessments made by an AI assisted CCTA with level 3 expert CCTA readers. In this multicenter international study, patient history data (BMI, age, sex, smoking history, diabetes, etc) were combined with a deep convolutional neural network AI (Cleerly) labelled CCTA images in order to estimate stenosis percentage, plaque volume, composition and presence of high-risk plaque. Results of this 232-patient study showed 99.7% accuracy, 90.9% sensitivity, 99.8% specificity, 93.3% positive predictive value and 99.9% negative predictive value for stenosis > 70%. When stenosis > 50% is considered, 94.8% accuracy, 80.0% sensitivity, 97.0% specificity, 80.0% positive predictive value and 97.0% negative predictive value was shown. When the expert reader comparison was performed for maximal diameter stenosis per vessel, −0.8% mean difference was found and for per patient comparison −2.3% mean difference was found. Reportedly, these excellent results show that the time-consuming expert reader evaluations could be processed much more quickly and cost-effectively by an AI [48].

Table 1 shows the detailed information about the outcomes of the reviewed publications which are applied for the diagnosis of the selected diseases with AI models and their performance summary.

## 4. Discussion

**Figure 2:**
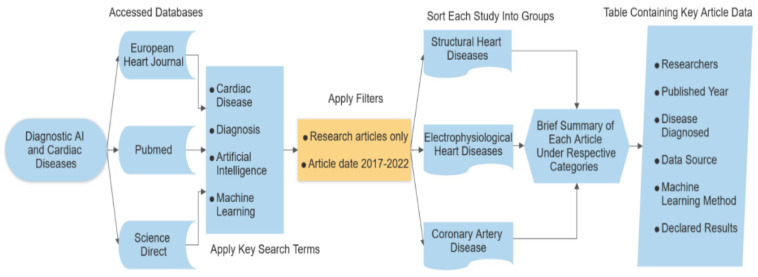
Flowchart of method design for this review.

**Figure 3:**
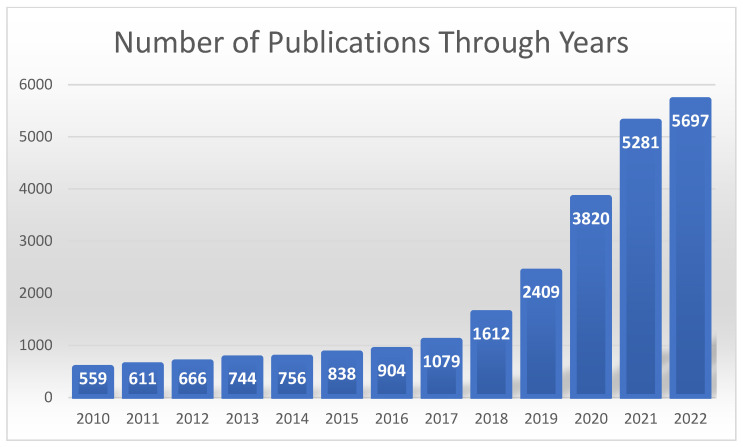
A bar chart showing the number of heart related AI/ML publications on Science Direct, Pubmed and European Heart Journal through years.

As it is evident from the number of articles reviewed above, overall publication of machine learning-related healthcare diagnosis research has been multiplicatively growing each year. By the same token, there is a good amount of research performed on structural heart diseases and especially on coronary artery disease. However, relatively, there are significantly less electrophysiology-based AI research publications on medical journal databases. Figure 2 shows the flowchart of the method design of this paper. The selected papers are limited to the years between 2017 and 2022. Figure 3 shows the yearly published heart-related AI/ML studies increased exponentially on Science Direct, Pubmed and European Heart Journal.

Similarly, a striking amount of machine learning research is being carried out that is focused on using echocardiograms and especially so with ECG signals. It is natural that one of the most useful pieces of data gathering tools in cardiology, ECG, is used as input in ML and especially in electrophysiology. However, usefulness of other forms of data, we believe, should not be underestimated. Hospitals with integrated patient health record systems have the ability to provide researchers with an immense amount of lab data containing a multitude of metabolic measurements, as well as patient profile and history records which is usually critical to how cardiologists normally perform their patient care routines. Although there are some studies that utilize these opportunities, we believe it has nowhere near reached its full capacity. On a similar note, the same is true for medical imaging as well, since one of the most routinely performed imaging tests is coronary angiography and there should be a well of untapped potential in that metaphorical alley.

It is clear that when looking at machine learning techniques, deep learning approach variations are much more preferred in general as opposed to classical single hidden layer supervised ML or unsupervised ML models. Convolutional neural networks, recurrent neural networks and cross-validation seems to be popular according to publications relevant to this review.

Overall level of success for reviewed AI diagnosis systems seems to indicate that they achieved noteworthy improvements on similar previous works or they are fresh and innovative ideas with a good starting performance. In some works, although negative predictive values were high, positive predictive values were comparatively very low, which indicates a need for further development and refinement.

In our opinion, however, one of the most important factors of true success in modern technology research is being slightly overlooked. We believe that a stronger collaboration between doctors and AI engineers should be present. Any research team of engineers should have at least a few specialists that they consult and, similarly, any research hospital interested in AI technology should employ the skills of engineers more often. It is not useful for a remarkable ML model, from a technical stand point, to be created and yet end up not being used because it does not realistically fulfil a need in healthcare. In order to create profound technological advancements, we believe, experts in the field of healthcare should identify the areas to work on and then engineers can direct their research efforts accordingly in order to build innovative support systems that are desperately needed.

Finally, we wanted to briefly raise questions about some ethical and legal considerations. First of all, what safeguards should be put in place in order to protect patient confidentiality since AI will be able to reach all data of all patients? What should we do about early risk assessments when the AI can tell us that a patient might be diagnosed with an illness in 5–10 years time? Which organization decides that an AI diagnosis system is deemed safe to use for patients; individual hospitals or governments? Who should be investigated when a malpractice case is brought up that involves an AI; doctors, hospitals, tech companies or engineers? Finally, what happens if doctors become, in two generations time for example, overly reliant on AI for diagnosis and their own judgement standards get lowered, or how can we prevent such an occurrence? At some point, we believe these questions will need to be answered.

## 5. Conclusions

The future of diagnostic artificial intelligence looks very promising, especially for diagnosis of cardiac diseases. Each year there are more and better research publications in AI and machine learning. Every new publication gets us one step closer to improving our current diagnostic tools and systems, providing us with a powerful ally in researching new approaches to understanding disease mechanisms and innovating new treatments for them. However, there are some ethical and legal considerations, as mentioned in discussions section, that will need to be seriously debated by all parties involved in the near future.

As it has been for decades before, contemporary computing power available in order to facilitate ML is one of the most obvious and important limitations for AI in our time. Sophisticated ML models that could be designed, and datasets that could be much larger and much more detailed would result in better overall performance. However, they cannot be employed unless there are sufficient strides in making the commercially available computing power on the market on par with the requirements of AI researchers.

It is also important to note that predictions and classifications made by AI models are only as strong as the dataset quality and selected feature reliability. Therefore, it is paramount for any AI system to be tested rigorously before being implemented. Another issue is that medical data are much harder to acquire compared to other fields. This is a considerable limitation since machine learning in general uses large amounts of data to train a model. Deep learning, a very promising subset of ML, is especially affected by this limitation since it has to utilize massive data sets in order to learn in a way that is a closer approximation to how humans learn.

Despite this limitation, there are a decent number of publicly available datasets like “CardioNet” by Ahn et al. [49], “Cardiovascular Disease dataset” from Kaggle [50] and “Heart Disease Data Set” from UCI Machine Learning Repository [51] to give a few examples, which somewhat alleviates this problem at least for now. On the other hand, since more sophisticated ML methods tend to require exponentially larger and more detailed datasets, in order to achieve the full future potential of AI assisted diagnosis, a much more comprehensive and meticulous solution needs to be implemented. This is the point where, we believe, the need for a standardized multi-national network of integrated healthcare system arises.

Apart from some of the obvious benefits of using previously performed tests from a hospital in order to reliably and easily get healthcare services from another hospital, the amount of data that can be obtained with such a system would be profound for AI researchers. Digitized medical imaging data, lab results, patient information and medical background, resulting diagnosis and short/long term prognosis would be obtainable for each patient, of course excluding patient identifier data, which means that any dataset that could be needed for any research can be extracted from this emergent database. This type of undertaking, however, would bring a new set of issues to be handled by international organizations where they would need to enforce ethical conduct and patient privacy, mediate a common data format, facilitate data sharing infrastructure and provide end user training.

In the end, we believe AI based diagnosis will not, and should not aim to, replace specialists and their roles in healthcare. ML technology should be an additional tool assisting doctors’ own judgement, save them and other healthcare professionals time on mundane and repeated tasks. Additionally, medical emergencies may benefit greatly from streamlined and fast decision-making processes where AI provides a lot of information to doctors with little data. Most importantly, AI end goal should be to provide better quality of healthcare, reduce hospital and administration costs to make getting medical help cheaper and more accessible, for everyone, everywhere.

## Figures and Tables

**Table 1:** Method and performance summary of reviewed publications.

Authors (et al.)	Year	Disease Diagnosed	Data Source	Machine Learning Method	Reported Results (Accuracy, Sensitivity, Specificity, PPV, NPV, AUC, F1)						
Hsiang [11]	2022	Low EF	Chest X-ray	Deep Learning						0.867	
Salte [12]	2021	LV dysfunction	Echocardiography	Deep Learning	GLS for: AI −12.0 ± 4.1%, Reference −13.5 ± 5.3%						
Bahado-Singh [13]	2022	Fetal congenital heart defects	Cell-free DNA	RF, SVM, DL		98%	94%			0.97	
Cheema [14]	2022	Heart Failure	IHS Data	Deep Learning	83%						
Shrivastava [15]	2021	Dilated Cardiomyopathy	ECG	N/A		98.8%	44.8%	1.8%	100%	0.955	
Kwon [16]	2020	Mitral Regurgitation	ECG	N/A						0.816	
Jentzer [17]	2021	LV dysfunction	ECG	N/A	76%					0.83	
Lee [18]	2022	LV dysfunction	ECG	Deep Learning	0.833	0.809		0.352	0.975	0.877	
Thalappillil [19]	2020	Aortic Annulus Size	Echocardiography	N/A	−4.62 to 1.26 mm difference for derived area and −4.51 to 1.45 mm for derived perimeter value						
Liu [20]	2022	Pulmonary Hypertension	ECG, TTE	Cross Validation DL		81%	79.6%			0.88	
Sun [21]	2021	Low EF	ECG, TTE	CNN Deep Learning	73.9%	69.2%	70.50%	70.1%	69.9%		
Thompson [22]	2019	Valvular, congenital	Digital Stethoscope	N/A	88%	93%	81%				
Harmon [23]	2022	LV dysfunction	ECG	CNN Deep Learning						0.93	
Makimoto [24]	2022	AV Stenosis	Digital Stethoscope	CNN Cross Validation	95.7%	97.6%	94.4%				0.93
Attia [25]	2022	Low EF	Digital Stethoscope	CNN						0.89	
Ghanayim [26]	2022	AV Stenosis	Digital Stethoscope	N/A		84%	92%				
Ueda [27]	2021	AV Stenosis	Chest X-ray	Deep Learning Ensemble	0.71	0.78	0.71	0.18	0.97	0.83	
Nakamura [28]	2021	PVC Origin	ECG	SVM, CNN	0.85						
Chen [29]	2020	AF	1-Lead ECG, PPG	CNN Deep Learning	93.27%	88%	96.41%				
Sau [30]	2022	AFL/SVT	ECG	N/A	86%						
Jo [31]	2021	Paroxisymal SVT	Sinus ECG	Deep Learning	0.97	0.868	0.972	0.255	0.998		
Chang [32]	2021	Arrhythmia	ECG	Recurrent NN	0.987					0.997	
Au-Yeung [33]	2021	Arrhythmia	ECG, BP, PPG	Random Forest		81.54%					
Lee [34]	2022	AF	Exercise ECG	Deep Learning	N/A						
Pandey [35]	2020	Arrhythmia	ECG	Ensemble SVM	94.4%						
Zhu [36]	2020	Arrhythmia	ECG	CNN							0.887
Otaki [37]	2022	CAD	SPECT	Deep Learning						0.83	
Braun [38]	2020	CAD	VCG	Supervised ML	82.5%	90.20%	74.4%				
Zhao [39]	2020	ST elevated MI	ECG	N/A	99.01%	96.75%	99.2%			0.995	0.937
Choi [40]	2022	ST elevated MI	ECG Image	N/A						0.919	
Cho [41]	2021	Plaque Type	IVUS	Deep Learning	96%	86%	97%				
Stuckey [42]	2018	CAD	CT	Supervised ML		92%	62%	46%	96%		
Cho [43]	2019	CAD	C. Angiography FFR	Supervised ML	81%					0.87	
Lee [44]	2021	CAD	Treadmill Test	RF, SVM, LR, K-NN, EGB		85%				0.74	
Lipkin [45]	2022	CAD	AI CCTA	N/A						0.88	
Kurata [46]	2019	CAD	CT FFR	N/A						0.907	
Tang [47]	2019	CAD	CCTA FFR	N/A	0.9	0.85	0.94				
Choi [48]	2021	CAD	AI CCTA	CNN Deep Learning	94.80%	80%	97%	80%	97%		

## Data Availability

The data is provided within the manuscript.

## Abbreviations

Abbreviations used in this article are listed below in alphabetical order.

AF	Atrial fibrillation
AFL	Atrial flutter
AI	Artificial intelligence
AUC	Area under curve
AV	Aortic valve
AV	Atrioventricular
BMI	Body mass index
BP	Blood pressure
CAD	Coronary artery disease
CCTA	Coronary computerized tomography angiography
CDC	Centers for Disease Control and Prevention
CNN	Convolutional neural network
CT	Computerized tomography
DL	Deep learning
ECG	Electrocardiogram
EF	Ejection Fraction
EGB	Extreme gradient boosting
FFR	Fractional flow reserve
GLS	Global longitudinal strain
HF	Heart failure
ICU	Intensive care unit
IHS	Integrated healthcare system
IVUS	Intravascular ultrasound
K-NN	K-nearest neighbor
LR	Logistic regression
LV	Left ventricule
LVEF	Left ventricular ejection fraction
MI	Myocardial infarction
ML	Machine learning
MPI	Myocardial perfusion imaging
NN	Neural network
NPV	Negative predictive value
PPG	Photoplethysmographic
PPV	Positive predictive value
PVC	Premature ventricular complex
QCT	Quantitative computerized tomography
RF	Random forest
SPECT	Single photon emission computerized tomography
SV	Supraventricular
SVM	Support vector machine
SVT	Supraventricular tachycardia
TAVI	Transcatheter aortic valve implantation
TET	Treadmill exercise test
TTE	Transthoracic echocardiography
VCG	Vectorcardiography
